# Strengthening community services to keep individuals with mental illness out of jail: a qualitative analysis of implementation mechanisms in 52 U.S. Counties

**DOI:** 10.1186/s40352-025-00390-0

**Published:** 2025-11-27

**Authors:** Jennifer E. Johnson, Maji Hailemariam, Faye Taxman, Benjamin J. Mackey, Hiywote Eshetu, Jiaxin Wei, Olzhas Zhorayev, Neelam Shukla, Niloofar Ramezani, Rochelle Rosen

**Affiliations:** 1 Charles Stewart Mott Department of Public Health, Michigan State University, 200 East 1st Street, Flint, USA; 2 Center for Advancing Correctional Excellence, Schar School of Policy & Government, George Mason University, 4400 University Drive, Fairfax, VA USA; 3 Department of Biostatistics, Virginia Commonwealth University, Box 980032, 830 East Main Street, Richmond, VA USA; 4 Center for Behavioral and Preventive Medicine, The Miriam Hospital, Providence, RI USA

**Keywords:** Implementation, Mechanism, Jail, Mental health, Substance use, Outcome, Community, Qualitative

## Abstract

**Background:**

Identifying mechanisms of implementation approaches helps improve them. This qualitative analysis examined how hypothesized mechanisms (performance monitoring, interagency teams, common goals/mission across agencies, and system integration) influenced implementation outcomes (number of evidence-based services [adoption], people served [reach], and resources to support evidence-based services [component of sustainment]) in county-level efforts to improve community services to keep people with mental illness out of jails.

**Methods:**

Our study statistician chose a representative sample of 60 U.S. counties and respondents from a sampling frame consisting of community mental health, community substance use, jail, and probation administrators in 950 U.S. counties. We reached and interviewed representatives from 52 of 60 counties (68 respondents in 52 interviews). Interviews were recorded, transcribed, de-identified, and coded using thematic analysis with deductive codes. Study team members created analysis summaries for each mechanism, which were reviewed and verified by other team members.

**Results:**

Administrators viewed all hypothesized implementation mechanisms as helpful for at least one outcome. They viewed performance monitoring (tracking metrics over time) as helping counties obtain resources to provide services, re-allocate existing services to better meet clients’ needs, and provided feedback needed to improve services. Interagency teams (i.e., regular meetings with multiple agencies) increased integration and continuity of care across treatment and jail/probation settings, improved client mental health and substance use outcomes, provided access to additional trainings and programs, and helped to change attitudes and policies to support evidence-based practices. Agreement on goals and mission across agencies improved availability of evidence-based practices, client outcomes, and collaboration between community providers and criminal justice partners, and facilitated interagency problem-solving. Integration of care across agencies helped get clients faster, more appropriate care, eliminating unnecessary frustration, illness, incarceration, and suffering. The presence of one mechanism tended to support use of other mechanisms. Representatives from small and medium/large population counties viewed mechanisms as similarly helpful. However, medium/large counties were often further along enacting the mechanisms.

**Conclusion:**

Respondents described performance monitoring, interagency teams, common goals across agencies, and system integration improving number of evidence-based services, people served, and resources for improving community services to keep people with mental illness out of jails.

## Contributions to the literature

- One way to improve implementation approaches is to identify how they work (i.e., implementation mechanisms).
- We know little about mechanisms of implementation efforts that cross systems with multiple goals, where health goals may be secondary.
- This study found that performance monitoring (tracking metrics over time for quality improvement), interagency teams, common goals and mission across agencies, and system integration were helpful mechanisms in cross-agency efforts to strengthen community services that keep individuals with mental health and substance use disorders out of jails.
- These mechanisms may also be useful in other cross-agency implementation efforts.

## Background

More than 7 million adults are arrested and enter the United States criminal justice (CJ)^1^ system, including pretrial detention, jail, probation, and parole, each year (Korhonen, 2024). CJ systems are low-resource settings that serve people with complex health and social needs. Counties (which often operate jails, community supervision such as probation and parole, community mental health, and substance use treatment systems) care for most individuals interacting with CJ systems; fewer people are sentenced to state or federal prisons. Counties are often overwhelmed by limited community services and overuse of jail incarceration for those with mental health and substance use disorders. Service improvement in the community (and not just in jails) is important to reduce the overuse of jail for mental health problems. Models for preventing CJ involvement due to mental health or substance use problems start with community crisis care (Substance 2020). Our recent study found that lack of available/affordable mental health and substance use services in communities, and not violent crime, predicted per capita county jail populations, making access to community care vital (Ramezani et al., 2022).

The National Association of Counties, the Council of State Governments Justice Policy Center, and the American Psychiatric Association Foundation developed the Stepping Up Initiative (American, 2020). The goal of Stepping Up is to reduce the overuse of jails for individuals with serious mental illness by improving access to community mental health services for currently or potentially justice-involved individuals. Counties join by passing a resolution to reduce unnecessary use of jail and increase access to community behavioral health services using a broad, locally adaptable six-step plan. Steps include regularly convening representatives of multiple agencies, collecting and reviewing data on individuals interacting with CJ systems, examining treatment and service capacity, developing a plan with measurable outcomes, implementing the plan, and tracking ongoing progress with data (American, 2020).

### Implementation mechanisms

Implementation approaches are collections of strategies used to prepare for, implement, and/or sustain a new policy or practice. Stepping Up can be conceptualized as an implementation approach because it seeks to increase use and availability of evidence-based mental health services. Understanding how implementation approaches result in outcomes, or the study of implementation mechanisms, is important for creating and improving implementation effectiveness (Lewis et al., 2018). The Implementation Mechanisms for Justice and Behavioral Health (IM Behavioral Health) Study (Johnson et al., 2021) conceptualized the Stepping Up steps as working through four implementation mechanisms (performance monitoring, interagency work groups, goal and mission setting across agencies, and system integration) to produce desired implementation outcomes (Table1) (Johnson et al., 2021), helping health and CJ agencies work together to improve mental health and substance use services county-wide (Taxman & Belenko, 2011). The study team chose these mechanisms based on their Criminal Justice Interagency Implementation Model (Taxman & Belenko, 2011) and subsequent CJ implementation work. Stepping Up was an independent ongoing natural experiment by which to examine these implementation mechanisms in action (see Johnson et al.^6^ for more details on theory). Hypothesized mechanisms of systems change involving both criminal justice and community treatment settings are described below.

**Table 1:** Hypothesized implementation mechanisms and implementation outcomes (Johnson et al., 2021)of efforts to reduce overincarceration of people with mental illnesses (American, 2020),according to the I.M. Behavioral health study

Construct
**Hypothesized implementation mechanisms**
Use of/capacity for performance monitoring
Use and functioning of interagency teams
Common goals and mission across agencies
System integration (vs. single programs)
**Implementation outcomes reflect county capacity to identify individuals with mental health or substance use disorders who are justice-involved and provide them with appropriate community care**
Number of evidence-based services offered [reflecting adoption]
Number of justice-involved clients receiving mental health and/or substance use services [reflecting reach]
Resources for evidence-based mental health and substance use services [one component of sustainment]

#### Performance monitoring (Johnson et al., 2021)

Performance monitoring is the *repeated*collection and use of metrics to assess population needs, choose next steps, and monitor progress. This is more than describing a population; it means following important benchmarks over time to guide decision-making and judge effectiveness of efforts. For example, counties might regularly monitor numbers of individuals with mental illness booked into jail to examine impacts of diversion efforts. They might monitor percent of individuals linked to community treatment after release from jail to examine effects of referral efforts. Such monitoring facilitates quality improvement, allowing systems to determine whether their efforts are having desired effects. For example, the city of Philadelphia published a good example of performance monitoring, showing how they tracked average daily jail census monthly over four years to examine iterative effects of a series of efforts to reduce their jail population (First, 2015).

#### Use and functioning of interagency teams (Johnson et al., 2021)

Mental health, substance use, jail, and probation agencies must cooperate and function as a team to address larger, systemwide issues. The need to work together across sectors (e.g., inpatient, outpatient, specialty care; mental and physical health; healthcare and social determinants) to optimally help patients is required in many settings other than CJ, including mental health, maternal health (National, 2024), and stroke rehabilitation (American, 2024). Interagency teams take different forms, but many rely on regular meetings where multiple agencies engage in collaborative information sharing and problem solving (Mackey et al., 2025).

#### Common goals and mission across agencies (Johnson et al., 2021)

It is difficult for agencies to work together to address common problems if they do not have a shared mission, and goals. Evidence-based health practices are more likely to be implemented in systems with clear, visible goals (Taxman et al., 2007; Melnick et al., 2009), where the practices are consistent with the agency’s mission (Taxman & Belenko, 2011; Friedmann et al., 2007). Agencies such as jails, police, and probation have primary public safety goals, with some secondary public health responsibilities. To achieve mental health and substance use goals county-wide, CJ and community systems need to agree that improving mental health and substance use benefits public safety (Taxman & Belenko, 2011). They also need buy-in of external stakeholders such as policymakers, police, judiciary, and voters.

#### System integration (Johnson et al., 2021)

Many agencies are involved in mental health and substance use care for individuals interacting with CJ. These include community mental health centers, substance use treatment agencies, police, courts, jail, probation, and parole. Service linkage among them is challenging and often inadequate (Henderson et al., 2009, 2008). Implementation models for these systems (Taxman & Belenko, 2011)hypothesize that their efforts will be most effective when they broaden engagement and ownership across agencies to develop a county-wide system of care (e.g., use of common screening tools or ability to move clients to where they might be better served), rather than adding a single program or staff training. Unfortunately, efforts to bridge fragmented systems of care for individuals interacting with CJ tend to implement new programs rather than working toward a cohesive system of care (Taxman & Bouffard, 2000; Henderson & Taxman, 2009).

### Rationale and study aims

The IM Behavioral Health Study (Johnson et al., 2021)is evaluating these implementation mechanisms and associated outcomes using Stepping Up, a national initiative to keep individuals with mental illness out of jail. The study involves longitudinal quantitative surveys from 950 U.S. counties and qualitative interviews with a representative subset of these counties. Although reform rhetoric is common, there are few rigorous, prospective studies examining implementation mechanisms and their relationship with behavioral health implementation outcomes in CJ settings. IM Behavioral Health is the first prospective national study evaluating mechanisms (or outcomes) of efforts to help justice and community behavioral health systems work together to keep individuals with mental illness out of jail. This information is critical to improving behavioral health outcomes and improving stability of living conditions for millions of the U.S.’s most vulnerable. The national Stepping Up Initiative, which seeks to change the interface between behavioral health services and the CJ systems provides a large natural experiment and a unique research opportunity (Johnson et al., 2021).

Findings also have novel implications for implementation science. Most implementation studies have been conducted within a single kind of organization (e.g., health systems or CJ agencies) rather than across networks of disparate agencies with differing goals, where some goals may be secondary (Lewis et al., 2018) (such as health goals in CJ agencies). The ongoing IM Behavioral Health Study seeks to identify target mechanisms in complex, multi-sector implementation efforts.

Aims of the overall IM Behavioral Health study (Johnson et al., 2021)are to: (1) examine implementation mechanisms of Stepping Up (using 475 Stepping Up counties and 475 control counties); (2) compare implementation outcomes of Stepping Up and control counties; and (3) use qualitative data to enrich an understanding of how the hypothesized mechanisms and how they produce outcomes. Previous papers (Mackey et al., 2025; Hailemariam et al., 2025) described counties’ processes of trying to engage the hypothesized mechanisms using the study’s Wave 1 qualitative data. The current article reports a planned analysis of Wave 1 qualitative data examining: (a) whether and how each of the hypothesized mechanisms produced outcomes; (b) how they influenced each other; and (c) any differences in how the mechanisms worked between small (population < 250,000) and medium or large (population >250,000) counties. Specifically, this article reports qualitative analysis of the relationships between implementation mechanisms and improvements (or lack thereof) in county-wide services (including number of evidence-based services [adoption], number of people served [reach], and resources to support needed services [one component of sustainment]).

Data come from interviews with 68 agency (community mental health, community substance use, jail, and probation) leaders in 52 representative U.S. counties. The analysis informs change efforts in systems that serve millions of the nation’s most vulnerable.

## Methods

The IM Behavioral Health Study (Johnson et al., 2021)is longitudinal, quasi-experimental study using surveys and interviews with community mental health, community substance use, jail, and probation leaders in 950 U.S. counties. It explores implementation mechanisms in efforts to reduce the number of people with mental illness in jail by improving community services, using Stepping Up as an example. Half the counties had enrolled in Stepping Up and half are matched comparison counties (Johnson et al., 2021). This analysis used data collected in the study’s baseline qualitative interviews conducted between December 2020 and March 2022. Because the goal of this analysis was to explore the relationship between hypothesized implementation mechanisms and outcomes (rather than to explore interventions that engage the mechanisms), this analysis did not distinguish between Stepping Up and matched comparison counties.

### Sample and procedures

The study team contacted community mental health, community substance use, jail, and probation leaders in 950 counties across the U.S. to participate in a survey (see Johnson et al (Johnson et al., 2021) for details of the survey methodology). The study statistician randomly selected a subsample of 60 counties (30 Stepping Up counties and their matched comparison counties) for qualitative interviews. The 60 counties were representative of the U.S. in terms of county size and reflected varying geographic regions.

We randomly assigned the primary contact agency for each county pair to be a community treatment (i.e., mental health or substance use) or correctional (jail or probation) agency for each county. Because Stepping Up focuses on keeping individuals with mental illness out of jail, we typically reached out to community mental health agencies or jails first. Following the Tailored Design Method protocol (Dillman et al., 2014) for maximizing response rates, we sent an initial email, with a follow-up email each week for 2 weeks. If we did not receive a response, we then called the potential respondent. If we did not receive a response, we approached the next agency in the county. The initial email described the study as “an evaluation of county-level efforts to reduce the use of jail for people with mental illness” to learn about barriers, facilitators, and practices on the ground in these efforts. The initial email emphasized “You have valuable perspectives to share that can help other counties.” We were able to contact leaders in 52 of the 60 identified counties for qualitative interviews.

This study was declared exempt by the Michigan State University Biomedical and Health Institutional Review Board (STUDY00003526). Consent for participation in the study was obtained using an electronic informed consent form.

In-depth interviews took place via videoconference. Interviews typically occurred with an interviewer, a note-taker, and one participant. However, several participants brought additional members of their agency and/or county to the interview. Therefore, our 52 county-level interviews included 68 total respondents. Questions addressed current mental health services in the county, county change processes, critical incidents, and the presence/absence and effects of the hypothesized implementation mechanisms. Mechanisms and outcomes are listed in Table 1. The interview introduction emphasized that there were no right or wrong answers and that we were seeking to understand their experiences. Interview questions were worded in a neutral way to reduce socially desirable responding (Table 2).

**Table 2:** Qualitative coding definitions

Hypothesized mechanism	Subcode	Additional definitions provided for coders	Related interview questions
Performance monitoring	Code 7c. Effects of data/performance monitoring on services. Processes by which data/performance monitoring did (or did not) lead to implementation outcomes listed in Table 1.	This is the use of data provided regularly (e.g., monthly, quarterly on things like number of people admitted, number with serious mental illness, costs, recidivism) to calibrate system decisions and help with system improvement.	Tell me a little about how your organization uses data to make decisions about services for justice-involved individuals.* What data do you use? How do you get these data? How do you use the data? (And what effects does that have?)
Interagency teams	Code 8c. Effects of interagency teams on services. Processes by which interagency teams did (or did not) improve implementation outcomes listed in Table 1.	This is more than just agencies collaborating. Teams implies meeting regularly to advance mutual goals and initiatives. This is typically more than 2 agencies, but 2 agencies can count if they meet regularly to advance mutual system goals and initiatives to improve care overall (e.g., create a mental health court or work on system design, not just coordinate care for individuals).	*How do you work with other organizations and agencies in your area (such as mental health*,* substance use*,* jail*,* probation*,* parole*,* county government) to improve mental health and substance use services for justice-involved individuals? What works well? What could work better? (And what effects does that have?)*
Agreement on goals and mission	Code 9c. Effects of cross-agency agreement on goals/mission on services. Processes by which cross-agency agreement on goals/mission did (or did not) improve implementation outcomes listed in Table 1.	The extent to which jail, community mental health, probation, substance use, and other agencies see eye to eye and agree on what needs to happen to improve services.	*To what extent do you see eye-to-eye with these other agencies about what needs to happen? Could you give me some examples? (And what effects does that have?)*
Focus on a system of care vs. adding a single program	Code 10c. Effects of focusing on a system of care (vs. a single program) on services. Processes by which focusing on a system of care (vs. a single program) did (or did not) lead to implementation outcomes listed in Table 1.	Typically involves > 2 agencies. Is more than just coordination of care. It is about creating an entire system of care in the county, so that any agency can get a person to where they need to go in any other agency. It’s about creating ways to bridge all the systems together, rather than just adding a single program (like care coordination).	*To what extent is mental health care for justice-involved individuals in your county integrated across agencies? Tell me a little about that. How does it work? Probe: can you give me an example of when integration across agencies has worked well? What has been most helpful or least helpful to changing it? (And what effects does that have?)*

*We defined “justice-involved individuals” as anyone interacting with 911 calls, local law enforcement, pretrial jail detention, court appearances, specialty courts, jail sentences, probation, and parole. These individuals may be in jail or in the community

**We also coded relevant responses from more general interview questions, such as “Tell me a little about how mental health and substance use services for justice-involved individuals are structured in your area” or “Tell me the story of how mental health services for justice-involved individuals in your area have improved (or not) over the past few years” or “Is there anything else we should know?”

### Data analysis

Qualitative interviews (*n*= 52) were recorded and transcribed by a professional transcription service. Transcripts were cleaned, reviewed for accuracy, and de-identified by the study team. We developed a priori qualitative codes from IM Stepping Up hypotheses about implementation mechanisms (Johnson et al., 2021). Each implementation mechanism had its own code. Under each mechanism code, subcodes reflected: (a) the current state of each mechanism (performance monitoring, interagency teams, agreement on goals and mission, and system integration) in the county; (b) how things came to be that way; and (c) effects of the mechanisms. This analysis used the “c” (effects) codes. Each transcript was coded independently by two trained coders using NVivo 12. All transcripts were coded independently by two coders, working in three teams. The coders met to review their coded transcripts together and worked to establish consensus. Using applied thematic analysis (Guest et al., 2011), the coders used the codes to label passages within the transcripts. After all the transcripts were coded, data were extracted and the team created summaries for each hypothesized mechanism reflecting: (a) the current state of the mechanism; (b) how it came to be that way; and (c) effects of the mechanism, by county size. Summaries were reviewed and verified by other team members (see the “about the authors” section at the end of the article for a discussion of reflexivity).

We compared mechanisms by county size rather than by Stepping Up status for two reasons. First, our previous analyses found that county size (i.e., population under or over 250,000) is a driving factor in the availability, structure, and functioning of community mental health, community substance use, and CJ services and resources available to integrate them (Zhorayev et al., n.d., Ramezani et al., 2022, 2023, Cuellar et al., 2022; Mackey et al., 2024, Taxman et al., 2025). Second, in a classic mediation model (where the intervention [vs. control] engages the mediator and the mediator predicts the outcome), the relationship between the mediator and the outcome is in theory similar for both conditions. The difference is that the intervention engages the mediator more than the control condition does.

In the results, speakers were identified by sector (mental health, substance use, jail, or probation), county size (small [population < 250,000] or medium/large [population > 250,000]). Results describing effects of mechanisms (Sect. 1 below) list number of references and number of counties. Descriptions of mechanisms’ effects on each other (Sect. 2) were summarized from a subset of the references in Sect. 1 that were double coded with how other mechanisms came to be. Comparisons of small/medium vs. large counties (Sect. 3) describe comparisons among subsets of the references described in Sect. 1.

## Results

Our 52 county-level interviews included 68 respondents. Of the 52 primary interviewees, 18 were from jails, 3 from probation, 22 from community mental health, and 9 from community substance use agencies, with representatives from other agencies added to interviews as the interviewee saw fit. Reflecting the United States, 75% of the 52 counties (*n* = 39) were considered “small” (population < 250,000) and 25% (*n* = 13) were medium/large (population > 250,000). To ensure representativeness of highlighted results, our 9 quotes come from 9 different counties, representing at least 14 respondents overall. Results reflected: (1) effects of mechanisms on implementation outcomes; (2) effects of mechanisms on each other; and (3) comparisons of small vs. medium/large counties. The Standards for Reporting Qualitative Research checklist was included as supplemental material.

### Effects on implementation outcomes (number of clients served, EBPs available, and resources for EBPs)

#### Effects of performance monitoring on implementation outcomes (37 references from 26 counties)

Respondents described performance monitoring as having three main beneficial effects: *obtaining needed resources*,* re-allocating existing resources to better meet needs*,* and improving their services to better meet their clients’ needs*, with only one counterexample (see end of section below). In terms of *obtaining needed resources*, respondents across agencies indicated that data was helpful in obtaining grant funding. Jail and mental health respondents described how data helps them communicate the relevance of mental health and other services to voters and elected officials to secure appropriations for programs. In one conversation, respondents explained:

*Speaker 1: [Data] helps us acknowledge that… mental illness is a real thing. It’s a problem. It helps us explain to the community, the prison board, the commissioners that this is what our staff is dealing with, and these are the reasons why.*

*Speaker 2: Why the county is funding this.*

*Speaker 3: When we went to [a mental healthcare vendor]*,* it was a big deal. Because [the new contract] had a big sticker price. [Our data] helps to identify to the commissioners*,* the taxpayers*,* the prison board that the investment is well spent because of what [the mental health vendor] brings to the table and the care. Just the identification process that they can provide to tell us about our inmate population so we have less liability*,* less lawsuits. *Conversation among jail respondents (small county).

Another county used performance monitoring to forecast future housing demand for individuals being released from jail, which led to the initiation of a new housing program.

Respondents also described using performance monitoring or data-driven decision-making to *re-allocate existing resources* to better meet needs. One mental health respondent from a medium/large county provided several examples of how disaggregated data analysis by race, zip code, and program enrollment helped address patients’ needs by locating service centers in high need areas of their city, restaffing the centers with culturally matched staff, and training staff to respond sensitively to trauma. These service improvements led to an improvement in customers’ trust indicators, and an increase in life satisfaction scores.

Data monitoring led another county to reallocate psychiatric coverage into their medical contract:

*Looking at the number of individuals coming into the Detention Center who needed to be seen by a psychiatric prescriber*,* we realized that you really do need to have a much greater access to a prescriber than what we were providing*,* and*,* really*,* 24/7 access. Between our department and the sheriff’s office and reviewing the data*,* we made the decision to make that a more robust service within the Detention Center*,* and*,* ultimately*,* part of their medical vendor contract. That was a decision that was data driven and budget driven… is it more costly for us to hire someone full time to be on call or to roll it into a contract? *Substance use respondents (medium/large county).

Finally, respondents across agencies described using performance monitoring to make evidence-based decisions to *improve their services* and programs. Regular data collection was identified as helpful for: determining whether programs are effective; developing new programs; adjusting or eliminating existing programs; making decisions about medication supply; improving therapy provision; improving racial equity; and monitoring, predicting, and reducing recidivism. One team explained that data monitoring helped them discover that many people were coming back to jail because they did not have anywhere to live, leading to the launch of a returning citizens housing program. As a result of ongoing data, another county embedded drug and alcohol counselors in job and family and children services to connect parents who have lost rights to children due to addiction. Respondents from two different counties described stratifying their substance use treatment program outcomes data by race to examine equity. They discovered that Black participants did not improve as much and/or were not offered medication assisted treatment at the same rates as white participants. Both systems took steps to improve equity in their service delivery and demonstrated that those efforts were successful using ongoing data and performance monitoring.

Regular data collection had little effect if it was not reviewed and considered. One mental health respondent said that although their agency aspired to make data driven decisions, the state had so many legislative requirements for new services that the agency did not have time to respond to or consider data to improve their services. Similarly, a jail respondent explained that the jail did not use data to adapt their services or make programmatic decisions. Rather, they reported data “up the chain of command” to be “filed and available for whenever there’s a need to use them.”

#### Effects of interagency teams on implementation outcomes (44 references from 22 counties)

Respondents described interagency teams leading to increased *integration and continuity of care* across settings, including better referral to care, better continuity of care, increases in the number of individuals receiving care, and better rates of staying involved in care. Respondents reported that these improvements were facilitated by ongoing meetings and collaborations between CJ and mental health/substance use agencies, which facilitated new partnerships.

New collaborative activities resulting from interagency teams included mental health and substance use provider access to detention facilities, embedding mental health staff in CJ and other agencies, mobile crisis and overdose outreach teams, and a new halfway house with embedded mental health and substance use services. One respondent explained:

*We had the connections with the community health centers. We started talking about where do they struggle? Universally*,* they said*,* “Look*,* when someone comes in off the street*,* they’re homeless. They’re psychotic. They’re addicted. We don’t know what to do. We know how to help them if they’re a little more stable*,* but we don’t have those resources.” We… started using crisis mobile teams in their facilities.… It was a logical next step to just start embedding those care coordination*,* and crisis mobile staff just right there with them.* -Mental health respondent (medium/large county).

Ongoing interagency communication also facilitated information sharing between jails and community-based providers, which was especially useful for continuity of services including medication-assisted addiction treatment.

Respondents also described interagency teams resulting in improvements in many client outcomes, including *increased diversion from CJ to community providers*,* reduced recidivism*,* and reduced community substance use*. This occurred through increased communication across agencies, more multi-agency partnerships, and more community engagement. One respondent described interagency teams leading to success of their diversion efforts through: (1) creating a state-wide health information exchange system, allowing them to see who is connected to services and (2) embedding mental health and substance use staff at partner agencies such as the jail. Embedded staff at the jail interviewed individuals upon arrival and, if the individual was in community treatment, recommended diversion to community services.

Several respondents also stated that interagency teams resulted in improved resources to support EBPs, such as access to *new and better tools*, trainings, programs, and ability to share data. These included mental health trainings for police or jail staff, better access to clinical records across jail and community providers, more grant funding, better ability to track and understand data, more sharing of expertise and information about EBPs, and better screening and referral tools and processes.

Respondents described interagency teams as helping to *change attitudes and policies* to support evidence-based practices. That is, respondents felt interagency teams helped to gain buy-in on the importance of mental health from key stakeholders (including law enforcement leaders) and to change policies they felt were overly punitive and unhelpful. Interagency teams influenced attitudes and policies through better trainings (e.g., crisis intervention, training correctional officers how to respond to individuals with mental illness), often provided by partnering agencies, and involvement of key figures in the county (e.g., the sheriff). Respondents described increased community involvement and awareness:

*We’ve moved the needle with our commissioners*,* with our county manager*,* with our sheriff… We did an all-day summit around opioid use disorder and what its impact is on the county. All the county leadership was there. … The sheriff at the time was one of the most effective speakers. He stood there and said*,* “We’re not gonna arrest our way out of this*,* so how do we move the needle in other ways?”*

*You had real county leadership*,* whether it was department heads or the commissioners and the county manager*,* law enforcement*,* a lot of county leadership very much coming together to talk about all these issues and what are some of the best practices? What are ways we can get out of this? Those conversations*,* I think*,* really pushed this community in deeper ways. *Mental health respondents (small county).

There were no notable counterexamples (i.e., of interagency teams having deleterious effects).

#### Effects of having common mission and goals across agencies on implementation outcomes (23 references from 17 counties)

Respondents described how having a common mission and goals across mental health/substance use and CJ agencies improved *availability of evidence-based practices*, including diversion, *improved client outcomes*, *improved collaboration* between community providers and CJ partners, *and facilitated problem-solving*. There were no notable counterexamples to these findings.

In terms of *increasing availability of evidence-based practices and improving outcomes*, respondents from several counties agreed that having common goals and priorities across agencies facilitated a team-based approach wherein mental health/substance use agencies and law enforcement collaborated to divert individuals from jail to treatment. Agreement on goals and mission also resulted in training and action plans to help jails respond to incarcerated people in mental health crises without the use of force. Agreement also improved substance use outcomes (fewer relapses) due to a more flexible approach (i.e., not “all-or-nothing”) and through initiating a medication assisted treatment program. It created a close working relationship between community services and law enforcement, which improved outcomes for individuals by resolving their mental health and substance use issues more permanently, reducing repeat calls to first responders.

Several participants said agreement on common mission and goals promoted more *intensive and sustained collaboration* with CJ partners. This, in turn, led to outcomes like mental health and substance use staff access to the local jail, more requests for assistance with mental health and substance use calls, law enforcement sharing police reports, and even a collaborative effort to block marijuana retail facilities from opening. These efforts were often spurred by increasing agreement on the importance of addressing mental health, sometimes prompted by education from crisis intervention training. One respondent explained:

*Sometimes law enforcement isn’t as quick to jump on board with some of these things… Our Sheriff has been really open to and ahead of the curve with a lot of the mental health stuff and realizing at a minimum*,* it was an issue that he could at least help his staff feel less stress*,* and also help community members too. He was really on board with just diving in and being open to all of the suggestions from social services…. That open-mindedness*,* especially with the law enforcement side of things has been helpful for us. *Mental health respondents (medium/large county).

Additional benefits of common mission and goals included better communication with other agencies, which came from increasing knowledge and awareness of each other, and more leadership investment in addressing mental health and substance use issues without arrest.

Respondents explained that agreement on goals and mission helped with *problem-solving* in general:

*It’s…trying to find a way to make our systems fit together even though*,* sometimes*,* we do feel like square pegs in round holes. That can be a real challenge. Sometimes it’s like*,* “All right*,* but we have holes*,* so we can make the peg round if we want to.” Sometimes it does boil down to like*,* “Let’s try and find a creative solution because the actual thing that should make this an easy solution is not actually possible in law. We’re not changing the confidentiality law anytime soon*,* so let’s try and find a way to make this work. You need information. I have information. How can we share this in a way that protects my system and protects the confidentiality of the individual receiving service*,* but also meets your needs of what you need to best serve this person because you are also out for their best interest?” That’s all of our common goal*,* and I think that’s really the thing that helps us work together even when we do feel like we’re bumping up against a wall. Our goal is the best possible outcome for the people that we serve*,* and we’re all serving the same people. It really just does*,* sometimes*,* take a little bit of creativity and WD-40 to make it work. *Substance use respondent (medium/large county).

#### Effects of system integration on implementation outcomes (14 references from 12 counties)

Respondents explained that system integration results in *getting more appropriate care to those who need it and getting it to them faster*. When care is integrated, services become more available, timelier, and more appropriate. They almost universally saw better system integration as eliminating unnecessary frustration, illness, incarceration, and suffering for their clients. Respondents gave many examples of how a lack of systematic services made clients’ lives unnecessarily difficult and dangerous. One respondent explained:

*I think in terms of what works well*,* we have some individuals who communicate well and try to make sure that our clients have what they need and are connected with all the services that they need*,* and that that can happen as seamlessly as possible. What does not work well is that that should not be contingent on an individual being a good communicator. It should be a system that’s in place that works regardless of an exceptional case manager*,* a good case manager*,* who just does a good job. When then we lose a case manager*,* or that person goes to another agency and is working even somewhere else within our system*,* then we have a hiccup. That’s not okay for our clients. I think we could do better at making that part of the system*,* not just getting case managers and peer supporters trained up to do that well. *Mental health respondent (small county).

Many told stories of how the creation of a system of care in their areas led to improved mental health and substance use outcomes for individuals interacting with CJ systems, and better maintenance of treatment outcomes over time. One respondent described how a mental health/law enforcement co-responding team saved someone’s life. Respondents also gave several examples of situations where having a system of care between jails and community providers averted crises and saved lives, such as preventing suicide among someone being released from jail.

Respondents explained that having a system of care creates a county environment that is safer and more diversion-oriented for individuals with mental health issues. Respondents shared success stories of how system-wide collaborations among the jail, probation/parole, the courts, and providers resulted in the diversion of individuals that were struggling with mental health and substance use issues and reducing jail recidivism dramatically. This occurred through ongoing communication across agencies, embedding mental health and substance use staff with law enforcement teams, regularly assessing mental health and substance use needs of those passing through probation, and having probation and parole work closely with case management and Forensic Assertive Community Treatment programs. Another county set up a system where they have:

*Dedicated people that were not law enforcement officers*,* that were not detention officers*,* interview every single inmate that comes in to jail and find whatever risk factor that they have*,* whether it be substance abuse*,* mental health abuse*,* transitional housing*,* whatever it is*,* and then bringing a partner into the fold within the community that could address that risk factor*,* and then having a treatment plan written*,* agreed upon before their release and then connecting them to that care upon that release. By doing that*,* we lowered our recidivism rate [by more than half]. *Jail respondent (small county).

### Effects of mechanisms on each other

#### Effects of performance monitoring on other mechanisms

A few counties described how performance monitoring helped with system integration efforts by showing where people were waiting for services or dropping out of care (e.g., waitlists, length of stay), where better integration was needed, and whether integration efforts were working. A few counties also described how performance monitoring could influence interagency teams. For example, having data on high rates of recidivism or high prevalence of mental health conditions among individuals involved in jail and probation were “compelling,” and drove interagency efforts to address them.

#### Effects of interagency teams on other mechanisms

Rather than agreement on goals and mission being a prerequisite of successful interagency teams, respondents described interagency teams creating greater alignment among agencies through sustained communication, working together on common tasks, and better understanding each other’s systems (e.g., what jails or community mental health centers can and cannot do).

Interagency teams and collaboration were also among the most cited factors leading to county-wide system integration, including facilitating smooth re-entry processes for individuals being released from jail.

#### Effects of common mission/goals on other mechanisms

Several respondents described how common mission and goals improved system integration. For example, agreement on goals and mission leading to mental health responders being co-located with law enforcement or mental health and substance use providers embedded at a probation day reporting center.

Some respondents explained how having common goals helped interagency teams get work done. For example, one respondent felt that their partnerships owed their success to the shared goal—reducing crime—held by all members of the interagency team.

Finally, one county provided an example of how having common goals across agencies helped resolve data and performance monitoring challenges:

*We do have a long-standing agreement*,* an actual inter-local agreement*,* with the county jail. In addition to our liaisons*,* we provide funding that supports some of their program service staff… That began as a way of initiating a particular program that we run in the jail*,* but it was also a means of being able to bridge that information gap because program services staff are jail employees. They have access to information we don’t. Then we have our employees who have access to our information. If we can get everybody working together*,* we can get over the hump. *Mental health respondent invited by the jail to a jail interview (medium/large county).

#### Effects of system integration other mechanisms

One respondent (a substance use respondent from a small county) called system integration a “nice idea” that causes problems in practice. This person saw system integration limiting data-driven decision-making because forms changed when systems integrate, preventing access to past data.

### Patterns of responses in small (population < 250,000) versus medium or large counties (population > 250,000)

#### Performance monitoring

Both small and medium/large county representatives reported that data monitoring helped them secure or reallocate funding (both appropriations and grant funded), improve their services, and forecast future demand for new facilities. However, medium-large counties seemed to have more access to ongoing data than did small counties.

#### Interagency teams

Both small and medium/large county representatives highlighted better integration of services, continuity of care, and improvement of diversion efforts resulting from the better partnerships and communication that arose from the interagency teams. Small counties (but not medium/large ones) mentioned improvements in new trainings for staff, data sharing, and increased community awareness and involvement.

#### Common mission and goals across agencies

Small and medium/large counties had similar responses in terms of the effects of having a common mission and goals across agencies. They both described agreement on goals and mission leading to closer relationships with law enforcement and improved system integration. Respondents in both groups of counties also gave the specific example of having mental health or substance use practitioners co-located with CJ practitioners (e.g., probation, law enforcement).

#### System integration

Respondents from both small and medium/large counties described system integration as improving mental health/substance use outcomes, improving diversion, and averting crises. Medium/large counties seemed more likely to have thought they had achieved a system of care. Some small counties mentioned relying on personal and interagency relationships to overcome gaps in their service system infrastructures.

## Discussion

This qualitative analysis of interviews with mental health, substance use, jail, and probation leaders from 52 U.S. counties examined county leaders’ perceptions of whether and how hypothesized implementation mechanisms influenced county-wide mental health and substance use services that support keeping individuals with mental health problems out of jails. All hypothesized implementation mechanisms were viewed as helpful for at least one implementation outcome. Respondents described performance monitoring helping counties: (1) obtain resources to provide services, (2) re-allocate existing services to better meet clients’ needs, and (3) provided feedback needed to improve services. Interagency teams: (1) increased integration and continuity of care across settings, (2) improved client outcomes, (3) provided access to additional trainings and programs, and (4) helped change attitudes and policies to support evidence-based practices. Agreement on goals and mission: (1) improved availability of evidence-based practices and client outcomes, (2) improved collaboration between community providers and CJ partners, and (3) facilitated problem-solving. Integration of care helped get clients more appropriate and faster care, eliminating unnecessary frustration, illness, incarceration, and suffering. Implementation mechanisms also tended to enhance each other. Results may be relevant to other implementation efforts occurring across systems with multiple and potentially differing goals.

The United States’ ~2,882 small (< 250,000) population counties and the ~ 261 medium/large (>250,000) population counties often differ in resources for mental health, substance use, and other services (Ramezani et al., 2023, Ramezani et al., 2020). In this study, representatives from both viewed performance monitoring, interagency teams, interagency agreement on goals and mission, and system integration as helpful. Possible differences we observed were that: (1) medium/large counties may have been further along in enacting the mechanisms (such as having stronger performance monitoring or system integration in place); (2) some small counties described relying on personal relationships to overcome gaps in system integration.

Current results inform and extend previous findings from the I.M. Behavioral Health Study. For instance, cross-sectional quantitative analysis of Wave 1 data (Taxman et al., n.d.) found that counties that used performance monitoring and interagency teams offered a higher number of evidence-based mental health practices, controlling for county size and demographics (agreement on goals and mission and system integration were not directly tested). In the current qualitative analysis, respondents also reported positive results of performance monitoring and interagency teams, showing consistency between qualitative and quantitative findings for these mechanisms.

Although proposed mechanisms were viewed as producing positive outcomes, our previous qualitative analyses suggested that some are more difficult to enact than others. For example, in a focused analysis of counties’ use of performance monitoring in this dataset,^19^ respondents characterized their current level of data use as rudimentary. Many respondents viewed data use as a tool for coordinating individual-level health or legal service, rather than tracking system-level performance metrics. Most counties indicated that they would need more resources – such as infrastructure, staffing, data expertise, or technical assistance - to establish cross-agency data sharing, standardized data management systems, or dashboards for monitoring performance over time. Therefore, although performance monitoring is a cornerstone of quality improvement initiatives and was viewed as helpful by respondents, many counties would need additional resources or technical assistance to put it in place.

On the other hand, another detailed qualitative analysis of counties in this dataset found revealed that forming inter-agency teams is relatively easy, even in small and low-resource counties (Mackey et al., 2025). They found that interagency teams did not have to reach consensus on goals and vision before working together, as that could be built through collaboration. The current analysis found that respondents in small counties rely on personal relationships to secure services for clients in the absence of infrastructure support. Therefore, convening inter-agency teams is a practical and quick strategy that both improves county services and lays the groundwork for other hypothesized mechanisms (such as agreement on goals and mission and system integration).

### Implications for practice and policy

Results suggest that all these approaches (i.e., performance monitoring, interagency teams, interagency agreement on goals and mission, and system integration) may be beneficial. However, if counties must choose one as a place to start, we recommend regularly convening interagency teams, and using the teams as a place to advance efforts toward common goals, performance monitoring, and system integration. Interagency teams makes sense as a place to start because: (1) Mackey et al.^11^ found that agreement on goals and missions often followed, rather than led, convening effective interagency teams; and (2) counties can typically convene interagency teams more quickly than they can set up performance monitoring and integrate systems. Therefore, interagency teams provide a potent and achievable place to begin county-wide efforts.

Qualitative and quantitative analyses show that performance monitoring is helpful it is considered a key part of quality improvement efforts. However, many counties would still require additional resources or technical support to implement it effectively. Policies could provide counties with funding to build capacity for key tracking system metrics over time - such as service connections and length of stay - and by enabling data integration across health and CJ, as demonstrated in some model systems (Arias et al., 2023; Miller et al., 2024; The 2025).

### Strengths and limitations

Strengths of this study included a novel question, a large and representative qualitative sample of county leaders from across the country, and use of rigorous and replicable qualitative methods (i.e., large representative sample, validation of analyses, clearly scripted interview). Given our representative sampling, findings are transferrable and of interest and use to other U.S. counties. Limitations included relatively few probation respondents (*n* = 3), limiting generalizability to probation agencies across the country. Although infrequently used in qualitative research, member checking would have strengthened our conclusions by confirming they aligned with respondents’ views. Longitudinal analyses from the I.M. Behavioral Health Study are forthcoming, as are detailed descriptions of whether and how counties achieve cross-agency agreement on goals and mission and system integration.

## Conclusions

Counties carry the brunt of mental health, substance use, and other care for individuals interacting with CJ systems, including those in the community. Findings suggest that performance monitoring, interagency teams, common goals and mission across agencies, and system integration improve county capacity to identify individuals with mental health or substance use disorders who are justice-involved and provide them with appropriate community care. Doing so reduces reliance on jails, produces better client outcomes, and makes more efficient use of county resources. It would be ideal if counties could use all four of these approaches. However, if counties must choose one to start, we recommend regularly convening interagency teams.

## Data Availability

Qualitative transcripts in this project necessarily identify county-specific grants, systems and services which have the potential to identify speakers. Therefore, transcript data is not available, in keeping with the confidentiality agreements made to study participants during the consent process.

## Footnotes

1 Some health scholars now prefer the term “criminal legal” over “criminal justice” when referring to systems such as jails and probation, recognizing that justice is not always served in these systems. However, in our cross-sector work (such as the work described in the current article), our jail, probation, and other partners have been clear that they do not identify with the term “criminal legal,” that they find it alienating, and that it seems presumptuous for those outside criminal justice research and practice to rename their field without consulting them. Because our goal with this work is to help health and criminal justice agencies, researchers, and practitioners work together, we refer to our criminal justice partners by their preferred term, “criminal justice,” in this article.
